# MicroRNA and piRNA Profiles in Normal Human Testis Detected by Next Generation Sequencing

**DOI:** 10.1371/journal.pone.0066809

**Published:** 2013-06-24

**Authors:** Qingling Yang, Juan Hua, Liu Wang, Bo Xu, Huan Zhang, Nan Ye, Zhiqiang Zhang, Dexin Yu, Howard J. Cooke, Yuanwei Zhang, Qinghua Shi

**Affiliations:** 1 Hefei National Laboratory for Physical Sciences at Microscale, School of Life Sciences, University of Science and Technology of China, Hefei, Anhui, China; 2 Department of Urology, the First/Second/Fourth Affiliated Hospital, Anhui Medical University, Hefei, Anhui, China; 3 MRC Human Genetics Unit and Institute of Genetics and Molecular Medicine, University of Edinburgh, Edinburgh, United Kingdom; University of Nevada School of Medicine, United States of America

## Abstract

**Background:**

MicroRNAs (miRNAs) are the class of small endogenous RNAs that play an important regulatory role in cells by negatively affecting gene expression at transcriptional and post-transcriptional levels. There have been extensive studies aiming to discover miRNAs and to analyze their functions in the cells from a variety of species. However, there are no published studies of miRNA profiles in human testis using next generation sequencing (NGS) technology.

**Results:**

We employed Solexa sequencing technology to profile miRNAs in normal human testis. Total 770 known and 5 novel human miRNAs, and 20121 piRNAs were detected, indicating that the human testis has a complex population of small RNAs. The expression of 15 known and 5 novel detected miRNAs was validated by qRT-PCR. We have also predicted the potential target genes of the abundant known and novel miRNAs, and subjected them to GO and pathway analysis, revealing the involvement of miRNAs in many important biological phenomenon including meiosis and p53-related pathways that are implicated in the regulation of spermatogenesis.

**Conclusions:**

This study reports the first genome-wide miRNA profiles in human testis using a NGS approach. The presence of large number of miRNAs and the nature of their target genes suggested that miRNAs play important roles in spermatogenesis. Here we provide a useful resource for further elucidation of the regulatory role of miRNAs and piRNAs in the spermatogenesis. It may also facilitate the development of prophylactic strategies for male infertility.

## Introduction

miRNAs are the class of endogenous non-coding RNAs, 19 to 25 nucleotides in size, which can regulate gene expression at either the transcriptional or post-transcriptional levels. Many studies have shown that miRNAs play an important role in various cellular processes, such as growth, proliferation, differentiation and death [1]. However, biological functions of many miRNAs are largely unknown, particularly in human spermatogenesis.

Spermatogenesis is a complex process through which diploid germ cells proliferate and differentiate into haploid spermatozoa. Emerging evidence has shown that small RNAs are essential for spermatogenesis and male fertility [2], [3]. First, several expression profile studies using cloning or microarray approaches have demonstrated that numerous miRNAs are exclusively or preferentially expressed in the testis or male germ cells of human and mouse [4]–[9]. Second, miRNA expression pattern appears to be different between immature and mature testes [7], [10], [11]. Last, spermatogenesis is disrupted at the early stage of proliferation and/or differentiation in mice with conditional knock-out of Drosha or Dicer [12]. Additionally, several studies have found that some miRNAs participate in mammalian spermatogenesis. For example, miR-122a is predominately expressed in post-meiotic male germ cells and promotes the degradation of transcripts of transition protein 2 (TNP2), a post-transcriptionally regulated testis-specific gene that involved in chromatin remodeling during mouse spermatogenesis [5]. miR-383 is associated with male infertility and promotes testicular embryonal carcinoma cell proliferation by acting as a negative regulator of proliferation by targeting IRF-1 (Interferon regulatory factor 1) [13]. miR-372 and miR-373 can also promote the proliferation and tumorigenesis of primary human cells by neutralizing p53-mediated CDK inhibition, which possibly through the direct inhibition of tumor-suppressor LATS2’s expression [14]. miR-184 whose expression was restricted to the germ cells from spermatogonia to round spermatids is involved in the post-transcriptional regulation of mRNAs of nuclear co-repressor 2 (*ncor2*) in mammalian spermatogenesis [15]. Recently, it has also been shown that miR-18, a member of the mir-1 cluster of miRNAs, directly targets heat shock factor 2 (*hsf2*), a transcription factor involved in spermatogenesis [16]. All these studies suggest that miRNAs are involved in the regulation of gene expression during spermatogenesis.

PIWI proteins are the subset of the Argonaute proteins and expressed predominantly in the germline of various organisms. They are essential for germ cell maintenance and spermatogenesis in Drosophila and mammals [17]. MILI, MIWI, and MIWI2 are three mouse PIWI proteins that bind small RNAs of ∼24–31 nt termed as PIWI-interacting RNAs (piRNAs) [18]–[22]. Recent studies suggested that piRNAs and PIWI proteins also play crucial roles in spermatogenesis. In *Mili*-deficient mice, spermatogenesis is arrested at the pachytene spermatocyte stage [23], while, *Miwi2*-deficient mice display a defect in the early prophase of meiosis I and a marked and progressive loss of germ cells with age [24]. Previous studies indicating that miRNAs and piRNAs are required for normal spermatogenesis but detailed information on these RNAs in human testis are still not yet illustrated. A comprehensive profiling of testis-specific small RNAs will provide an insight into the mechanisms by which these small RNAs coordinate their target genes to regulate spermatogenesis and thus facilitate the understanding of causes for human male infertility. NGS has become increasingly popular in recent years because it can generate a massive amount of sequence data. It was shown to detect 25% more transcripts than microarray analysis [25]. NGS can successfully discover low abundance novel miRNAs due to its high sensitivity in detecting the genes with low expression levels than microarray [26]–[30]. NGS has revolutionized many aspects of genetic and biomedical research [31].

In the present study, we describe deep sequencing analysis of the small RNA transcriptome from testes of three normal men by using high-throughput Solexa technology. Out of the identified miRNAs, 770 have been reported by other investigators in human tissues other than testicular tissues and 5 have not been reported earlier. The expression of 5 newly identified miRNAs and 15 randomly selected already known miRNAs was validated by qRT-PCR. Notably, GO and pathway analysis of the potential miRNA target genes indicated that miRNAs are involved in many important functions in human testis, including the homologous recombination pathway. This study provides a useful resource to further elucidate the regulatory role of miRNAs and piRNAs in spermatogenesis and may facilitate the development of treatment strategies for male infertility.

## Results

### Sequence Analysis of Short RNAs

In order to obtain a comprehensive view of small RNAs expressed in adult human testis, we used Solexa deep sequencing technology for small RNA libraries prepared from three adult men. Total, 15,118,197 reads were obtained by sequencing. After removing 5′adaptor, trimming 3′ adaptor sequences, cleaning up contaminations and filtering out low quality reads, 14,608,234 reads were obtained, representing 4,818,130 unique tags (Table S1 and Figure S1). The majority of these unique tags were 22 nt in size, followed by 26–27 nt, which were consistent with the fragments of piRNAs (Figure S2). Among these unique tags, 5,568 corresponding to 3,840,018 reads and 25,845 corresponding to 1,051,404 reads were matched to known miRNA and piRNA sequences, respectively. The rest of the sequences were matched into other types of RNAs, including rRNAs, repeats and snRNAs (Table 1). The complete lists of miRNAs and piRNAs were shown in Table S2. All the data has been deposited in the ArrayExpress database (accession number: E-MTAB-1629).

**Table 1:** The match results of clean reads.

Category	NO. of Unique tags	Percent (%)	Total reads	Percent (%)
**miRNA**	5568	0.18	3840018	33.5
**piRNA**	25845	0.82	1051404	9.17
**rRNA**	44949	1.42	425714	3.71
**repeat**	1278335	40.41	2041160	17.8
**scRNA**	4858	0.15	13775	0.12
**snRNA**	9394	0.30	65787	0.57
**snoRNA**	2800	0.09	23848	0.21
**srpRNA**	840	0.03	3541	0.03
**tRNA**	8019	0.25	50549	0.44
**Unannotated**	1783143	56.36	3948377	34.44
**Total**	3163751	100.00	11464173	100.00

### Chromosome Location and Characteristics of Top Abundant Known miRNAs

**Figure 1:**
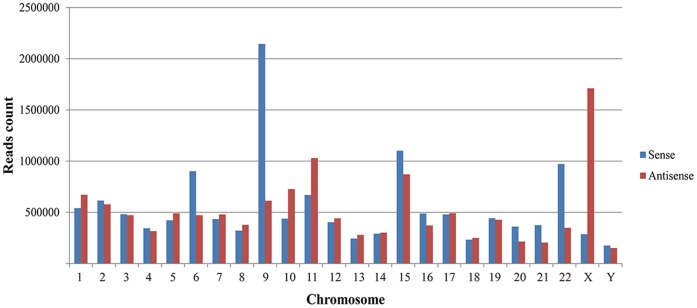
Number of small RNA tags that locate on each chromosome.

**Figure 2:**
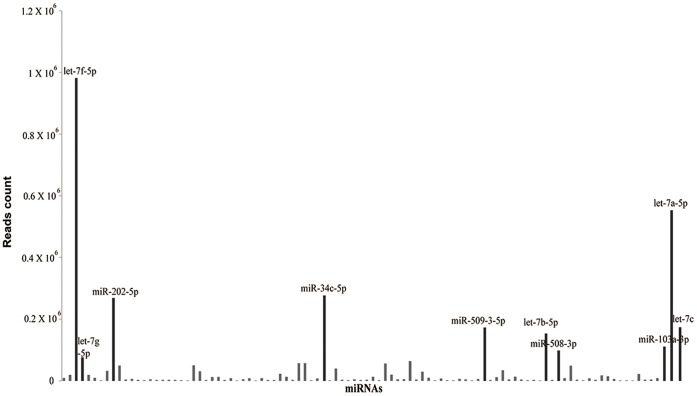
The expression profile of top abundant known miRNAs. Out of the 770 known miRNAs detected, the top 10 miRNAs were named.

**Figure 3:**
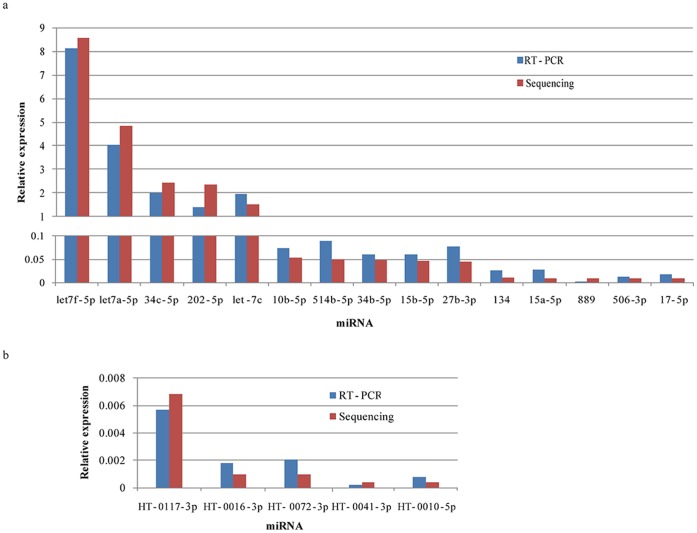
Confirmation of miRNA expression by qRT-PCR. a) Validation of the expression of fifteen known miRNAs belonging to three expression levels (high, median, low); b) Validation of the expression of the 5 novel miRNAs.

To understand the distribution pattern of all the reads on chromosomes, the location of each tag on chromosome was analyzed (Figure 1). Chromosome 9 harbors most of the reads, followed by chromosome X, 15, 11 and 6 respectively. Ten miRNAs have relatively more read counts, indicating that they are highly expressed in human testis (Figure 2). The location reads on chromosomes, unique tag counts and total read counts are shown in Figure 1 and Table 1. The top abundant miRNAs were mostly originated from chromosome 9 and X, followed by chromosome 11, 22 and 5 respectively. To validate expression of miRNAs detected by Solexa sequencing technology, fifteen known miRNAs representing three levels of expression were randomly chosen for quantification by qRT-PCR. The levels of the fifteen known miRNAs measured by qRT-PCR are consistent with the results obtained from Solexa sequencing technology, which indicated that the expression of miRNAs detected by Solexa sequencing is reliable (Figure 3a).

### Identification of Novel miRNAs

To identify more potential miRNAs in human testis, the unclassified tags were further processed by Mireap (http://sourceforge.net/projects/mireap). Only those tags meeting the default parameters and with read counts greater than 45 were defined as candidate novel mature miRNAs. On the basis of this analysis, 5 novel miRNA genes encoding 5 mature miRNAs were identified. Four out of these 5 novel miRNAs started with uridine or adenine at the 5′ end, a base that has been observed for miRNAs before. The generation of mature miRNAs derived from the same precursors has also showed heterogeneity, with more mature products from the 3′ ends than from the 5′ ends (Table S3). To confirm the expression of the novel miRNA candidates, aliquots of the RNA samples used for sequencing were subjected to the qRT-PCR assay with similar expression levels detected for all the 5 novel candidate miRNAs by the two techniques (Figure 3b).

### Human Testis Expresses Large Number of piRNAs

piRNAs (24–31 nt in length) are required for germline development in males and females [17], [32], [33]. A distinct subset of small RNAs with a peak size distribution of 25–28 nt was detected (Figure S2). After annotation analysis, most of these reads were matched to detected piRNA fragments with 20,121 piRNAs. The reads mapped to the piRNA catalog had a strong preference for the A at position 10, while those derived from antisense strands showed a preference for the U at position 1. These were completely consistent with the Ping-Pong model of piRNA biogenesis. The top 10 abundant piRNAs were extracted (Table 2), and they represent 61% of total piRNAs.

**Table 2:** The expression of top ten most abundant piRNAs.

NO.		piRNA			Most abundant tag			
	ID	Sequence	Length (nt)	Counts		Sequence	Length (nt)	Counts
1	DQ571592.1	AUCGGAACCUGCAGACACUCGUGGAGGCGUC	31	106965		UCGGAACCUGCAGACACUCGUGGAGGCGUC	30	77387
2	DQ601822.1	UAGGUGUGGAGCUUCCCGACCGGCUG	26	87638		UAGGUGUGGAGCUUCCCGACCGGCU	25	68013
3	DQ572237.1	UAUGAGCUUUAGAAUCAGUCAAGAGG	26	84463		UAUGAGCUUUAGAAUCAGUCAAGAGG	26	83929
4	DQ576939.1	UCGGAACCUGCAGACACUUGUGGAGGA	27	22571		UCGGAACCUGCAGACACUUGUGGAGGA	27	22190
5	DQ588372.1	UGGGAACGAGAAGACACUCAUGGAGG	26	22435		UGGGAACGAGAAGACACUCAUGG	23	8034
6	DQ588346.1	UGGGAACCAGAAGACACUCCUGGAGGAGUC	30	21052		UGGGAACCAGAAGACACUCCUGGAGGAGUC	30	20043
7	DQ601651.1	UAGGGACAAGAAGACACUCCUGCAGGAGUCGU	32	18391		UAGGGACAAGAAGACACUCCUGCAGGAGUC	30	17859
8	DQ569941.1	AAAGAAUGAAGAAGAACUUACUUGGCCU	28	16135		AAGAAUGAAGAAGAACUUACUUGGCCU	27	6886
9	DQ588403.1	UGGGAACGAGAAGACACUCGUGGAGGC	27	11005		UGGGAACGAGAAGACACUCGUGGAGGC	27	11004
10	DQ577923.1	UCUGCCUGAACUACACUGAGAAUGCAU	27	10495		UCUGCCUGAACUACACUGAGAAUGCAU	27	10436

### Prediction of miRNA Targets

In order to better understand biological functions of the most abundant known and novel miRNAs, putative target genes of miRNAs were predicted using miRanda program with the strict criteria described in Materials and Methods. The putative target genes of known miRNAs appeared to be involved in a broad range of biological processes with most of the target genes related to spermatogenesis, cell cycle and signal transduction pathways (Table 3 and Table S4). After GO analysis, KEGG was used to construct a pathway enrichment of predicted miRNA target genes. Many signaling pathways were found to be involved, including meiosis and p53 signaling (Table S5).

**Table 3:** The top 10 most enriched GO terms of biological processes, molecular functions and cellular components in predicted targets of 10 abundant known miRNAs.

Description of Go term	Human testis		Genome			
	Number	%	Number	%	E-ratio	p-value
**The top 10 most enriched biological processes**						
Regulation of ARF protein signal transduction (GO:0032012)	7	0.52	16	0.11	4.79	2.92E-04
Phospholipid transport (GO:0015914)	7	0.52	20	0.14	3.83	1.42E-03
Cytokine production (GO:0001816)	6	0.45	19	0.13	3.46	5.58E-03
Regulation of protein phosphorylation (GO:0001932)	8	0.6	28	0.19	3.13	2.87E-03
Negative regulation of phosphorylation (GO:0042326)	6	0.45	23	0.16	2.86	1.51E-02
Negative regulation of BMP signaling pathway (GO:0030514)	7	0.52	28	0.19	2.74	1.13E-02
Regulation of mitotic cell cycle (GO:0007346)	6	0.45	25	0.17	2.63	2.26E-02
Positive regulation of glucose import (GO:0046326)	6	0.45	25	0.17	2.63	2.26E-02
Gene silencing by RNA (GO:0031047)	8	0.6	34	0.23	2.58	1.02E-02
Sperm motility (GO:0030317)	7	0.52	30	0.2	2.55	1.66E-02
**The top 10 most enriched cellular components**						
Interleukin-6 receptor complex (GO:0005896)	2	0.13	3	0.02	7.32	2.34E-02
Senescence-associated heterochromatin focus (GO:0035985)	2	0.13	3	0.02	7.32	2.34E-02
Laminin-11 complex (GO:0043260)	2	0.13	3	0.02	7.32	2.34E-02
Cytoskeletal part (GO:0044430)	2	0.13	3	0.02	7.32	2.34E-02
Eukaryotic translation initiation factor 2 complex (GO:0005850)	2	0.13	4	0.02	5.49	4.40E-02
Microfibril (GO:0001527)	3	0.2	7	0.04	4.71	2.00E-02
Neuronal cell body membrane (GO:0032809)	3	0.2	7	0.04	4.71	2.00E-02
PTW/PP1 phosphatase complex (GO:0072357)	3	0.2	7	0.04	4.71	2.00E-02
Neuron projection terminus (GO:0044306)	3	0.2	8	0.05	4.12	2.99E-02
Voltage-gated sodium channel complex (GO:0001518)	5	0.34	14	0.09	3.92	6.24E-03
**The top 10 most enriched molecular functions**						
Phospholipid-translocating ATPase activity (GO:0004012)	6	0.41	15	0.09	4.41	1.36E-03
ARF guanyl-nucleotide exchange factor activity (GO:0005086)	7	0.48	18	0.11	4.29	6.56E-04
Neurotransmitter:sodium symporter activity (GO:0005328)	7	0.48	19	0.12	4.07	9.57E-04
Protein phosphatase inhibitor activity (GO:0004864)	6	0.41	21	0.13	3.15	9.15E-03
ATPase activity, coupled to transmembrane movement of ions,						
phosphorylative mechanism (GO:0015662)	9	0.62	37	0.13	3.15	9.15E-03
Galactosyltransferase activity (GO:0008378)	6	0.41	25	0.16	2.65	2.18E-02
Insulin receptor binding (GO:0005158)	7	0.48	31	0.19	2.49	1.90E-02
Rab GTPase binding (GO:0017137)	9	0.62	40	0.25	2.48	8.52E-03
Voltage-gated calcium channel activity (GO:0005245)	6	0.41	29	0.18	2.28	4.29E-02
Hydrolase activity, acting on acid anhydrides, catalyzing						
transmembrane movement of substances (GO:0016820)	9	0.62	45	0.28	2.21	1.83E-02

The putative target genes for the 5 identified novel miRNAs were also appeared to be involved in a broad range of biological processes (Table S6). Most of the enriched processes are metabolic, development and signal transduction (Table S7). According to KEGG pathway analysis of these putative targets, the homologous recombination pathway was enriched (Table S8 and Figure S3). Four members (*top3b*, *mus81*, *mre11a*, *nbn*) of this pathway were targeted by three novel miRNAs (HT-m0117-3p-*top3b*,

HT-m0016-3p- *mus81*, HT-m0072-3p/HT-m0041-3p/HT-m0010-5p- *mre11a*, and HT-m0010-5p- *nbn*), the detail description of the miRNA-mRNA pairs are shown in Table S9.

## Discussion

Infertility is a worldwide reproductive health problem which affects 10–15% of couples, about half of the cases are due to defect in male counterpart [34]. However, to date, only few contributing factors have been found to be associated with abnormal spermatogenesis. An increasing number of studies indicated that spermatogenesis is strictly regulated transcriptionally and post-transcriptionally while small RNA molecules are important regulators of gene expression at both transcription and post-transcription levels.

Generating expression profiles of small RNAs in human testis is a prerequisite for thorough understanding of their roles in the regulation of spermatogenesis. Deep sequencing techniques have resulted into a sharp increase in the number of novel microRNAs. Many miRNAs detected using NGS are able to be by microarray assays. For example, in a recent study using both NGS and microarray analysis to discover novel miRNAs in rat kidneys, the rno-miR-509-5p, rno-miR-509-3p and rno-miR-1306-5p that expressed at low levels were only detected by NGS, but not by the microarray [35].

Although human miRNAs have been studied extensively in the past several years, no systematic study has been reported on miRNAs profiling in human testis by using deep sequencing. In the present study, we used Solexa sequencing technology on small RNA libraries prepared from three adult human testes. Total, 15,118,197 reads were detected, out of which 14,608,234 were clean reads and represents 4,818,130 unique tags (Table S1 and Figure S1). From these unique tags, we identified 770 known and 5 novel miRNAs, which is significantly more than that obtained by using microarray analysis, where only 161 miRNAs and 120 miRNAs were detected in normal human testis in the previous two studies [36], [37]. This indicates that the NGS is definitely required to thoroughly characterize miRNA profiles in human testis.

RT-PCR was used to validate the already known and novel miRNAs the results showed that all the tested miRNAs were expressed in human testis (Figure 3a and 3b). It suggests that the miRNA profiles in normal human testis which we obtained by NGS, was reliable. Furthermore, the GO term annotation and the KEGG pathway analysis indicated that the target genes of the top abundant known miRNAs were involved in the meiosis pathway, which further demonstrates the reasonableness of our data. And for the 5 novel miRNAs, homologous recombination pathway was enriched based on the KEGG pathway analysis, the targeted genes (*top3b*, *mus81*, *mre11a*, *nbn*) are involved in DSB (double-strand break) repair in meiosis. As it has already been known that the DSB repair is an important process in spermatogenesis, Top3 and Mus81 are specific endonuclease, which can cleave stalled replication forks, allowing replication to continue [38]. MRE11, NBS1 and RAD50 form MRN complex which specifies both 3′-5′ exonuclease and single strand endonuclease activities, as well as limited DNA un-winding activity [39], [40]. All these results suggest that the dataset is reliable not only for characterizing expression profiles of known miRNAs but also for the discovery of novel miRNAs.

In agreement with miRNA microarray results showing high representation of *let-7* family members in human testis [4], we found that the most abundantly expressed miRNAs in human testis are let-7f-5p and its family members let-7a-5p, let-7c, let-7b-5p and let-7g-5p. Other miRNAs that were abundantly expressed in human testis include miR-34c-5p. Previous studies demonstrated that the miR-34 family (three highly related miRNAs–miR-34a, miR-34b, and miR-34c) are directly induced upon p53 activation in multiple cell types, and this miRNA family have been regarded as critical downstream effectors of p53 [41]–[48]. However, a recent study found that in mice with targeted deletion of all three members of the miR-34 family, the p53 response was not impaired in a variety of *in vivo* and *in vitro* assays, indicated that miR-34 members are not critical for downstream effectors of p53 [49]. Inhibition of miR-34c could prevent mouse male germ cell apoptosis through targeting ATF1 (activating transcription factor 1) [50], which mediates the transcriptional response of various extracellular signals and it is involved in cell viability and cell transformation [51]–[54], providing a novel mechanism with involvement of miRNAs in the regulation of germ cell apoptosis.

In the present study we found that miR-103a-3p was also abundantly expressed in adult human testis. Previous studies demonstrated that miR-103 was involved in various biological processis such as brain development, adipocyte differentiation, lipid metabolism, hematopoiesis, and immunity [55]–[58]. A recent study has reported that miR-103 also expressed in testis of piglets but not in adult pig testis, which indicates that miR-103 expression pattern depends on the developmental stages of pigs and may have important biological roles in testis [8]. miR-202-5p was highly expressed in adult human testis, which is consistent with the observation in mouse testis [6]. In chicken, the expression of miR-202 was observed to be sexually dimorphic, with up regulation in the development of testis from the onset of sexual differentiation. These findings indicated that miR-202 may function in the regulation of testicular development and spermatogenesis [59]. Interestingly, miR-508-3p, and miR-509-3-5p that have not been associated with spermatogenesis previously but these were found to be highly expressed in our dataset. A recent study demonstrated that miR-508-3p and miR-509-3p played an important role as tumor suppressor during tumor formation [60]. Overexpression of miR-508-3p and miR-509-3p could suppress the proliferation of renal cell carcinoma (RCC), induce cell apoptosis, and inhibit cell migration in vitro [60].

We have also identified a distinct subset of 25–28 nt long small RNAs expressed in human testis. In contrast to miRNAs, these piRNAs do not have a precursor with stem-loop, and do not require Dicer for their processing [61], suggesting that they have a different mechanism of biogenesis. The analysis of the reads mapped to the piRNA catalog suggested that piRNA biogenesis in adult human testis is completely consistent with the Ping-Pong model. piRNAs are expressed predominantly in the germline in mammals [18], [62], [63] including human beings. The mechanism of piRNA action has not been well interpreted in mammals. Several studies suggested that they play roles in repression of retrotransposons and post-transcriptional regulation by forming PIWI-interacting RNA complex (piRC) with PIWI proteins [20], [24], [62]. In our study, the targets for the most abundant piRNAs were predict using NCBI BLAST package and the detail information were summarized in Figure S4. As shown in Figure S4a, 74.93% potential targets appeared to be retrotransposons and the remaining 25% were repeat, mRNA and other-RNA. Futhermore, the principal classes targeted by piRNAs were Non-LTR (Figure S4b) and 92.4% Non-LTR were LI (Figure S4c). Future studies will help to elucidate the biogenesis and function of these novel piRNAs in spermatogenesis. Also, we found that twelve of the piRNAs with >1000 reads map uniquely using Blat within TDRG1(testis developmental related gene1) (Table S10), one locus corresponding to DQ601822.1 generates >118,000 reads (t0000196–t0000046), and some forming contigs (t0000248–t0000407). Several others mapped uniquely in a sense orientation to an intron of CYP19A1 (Table S11). TDRG1 is a developmentally regulated testicular-specific gene and CYP19A1 catalyses androgens into oestrogens [64]. Both of them play important roles in human spermatogenesis [65]. piRNAs map uniquely within these two genes suggest these piRNAs may play important role in spermatogenesis.

In mouse and human, a lot of miRNA genes usually present as clusters on different chromosomes [66], [67]. The present study demonstrated that the chromosome encoding the highest number of reads was chromosome 9, followed by chromosomes X, 15, 11 and 6. Some miRNA genes also appeared as clusters on chromosomes, for example, chromosomes X have 7 miRNA clusters, chromosomes 9 have 2 clusters and chromosomes 11 have 2 miRNA clusters. Each clusters had at least 2 genes, with miR-532∼miR-188 cluster in chromosome X having 2 genes, and mir-363∼106a cluster having 6 genes. However, the physiological significance of multiple miRNAs encoded by genes located within the same cluster remains unknown. One possibility is that they are derived from the same pri-miRNAs, which are transcribed under the control of the same promoters. The co-production of these miRNAs may also suggest that they are functionally related. By comparing the genomic structure of a miRNA cluster region on X-chromosome in human with four primates (chimpanzee, orangutan, rhesus macaque, and marmoset), researchers observed rapid sequence evolution of the miRNAs in this region, which highlighted important functions of these miRNAs in primates [68]. Song and his colleague demonistrated that about 86% of X-linked miRNAs actually escape meiotic sex chromosome inactivation (MSCI) during spermatogenesis, this unprecedented escape from MSCI suggested that they may have a critical function in spermatocytes from mid- to late pachytene stage of spermatogenesis, for instance, they probably participate in the process of MSCI, or essential for post-transcriptional regulation of mRNAs during late meiotic and early postmeiotic stages of spermatogenesis [69].

Another major advantage of the sequencing approach would be the ability to evaluate for possible miRNA editing events. David Bartel’s group described several mouse miRNAs that were edited to a significant degree in adult mouse brain and testis [70]. In our study, we also found some miRNAs from human testis with editing (Table S12). Furthermore, the differential level of the 3p (0.00043) vs 5p (0.00057) reads could be related to the degree of editing observed.

In summary, for the first time we have analyzed the known and novel miRNAs in human testis, by high throughput Solexa sequencing. Stem-loop RT-PCR analysis confirmed the expression of these miRNAs in the testis. The GO term and KEGG pathway annotations for the predicted miRNA targets further illustrate the likely roles of these miRNAs during spermatogenesis. This study provides a useful resource for further elucidation of regulatory role of miRNAs and piRNAs in spermatogenesis and may facilitate the development of therapeutic strategies for male infertility.

## Materials and Methods

### Testicular Sample Collection

Testicular tissues were collected from three cadaver (aged 28 to35 years) from the First Affiliated Hospital of Anhui Medical University, Hefei, Anhui, People’s Republic of China. Histological examination of all showed normal spermatogenesis. Each man has fathered at least one healthy child. All the procedures of sample collections and usages of human tissues were approved by the Institutional Review Board at First Affiliated Hospital of Anhui Medical University. Written informed consents were obtained from family members of the cadaver men prior the collection of tissue samples.

### Small RNA Library Preparation and Sequencing

Total RNAs were extracted from the human testis using TRIzol (Invitrogen). These samples were then subjected to 15% (w/v) denaturing PAGE (polyacrylamide gel electrophoresis) and the small RNA fragments of 18–28 nt were isolated. The small RNA molecules were ligated to 5′ and 3′ adaptors sequentially and then converted to cDNA by reverse transcription followed by PCR amplifications. Finally, approximately 20 µg of RT-PCR products per sample were sequenced directly using Solexa Genome Analyzer according to the manufacturer’s protocols.

### Small RNA Analysis

After removing 5′ adaptor sequences (GTTCAGAGTTCTACAGTCCGACGATC), trimming 3′ acceptor sequences (TCGTATGCCGTCTTCTGCTTG), filtering low quality reads (the quality value was calculated by Q = (ASCII character code) –64. If Q <10, the reads were defined as low quality reads) and cleaning up contaminated reads, the occurrence of each unique read was counted as tags. These unique tags were mapped to the human genome (GRCh37.p5) using SOAP2.0 [71]. Tags with perfect match or one mismatch were retained for further analysis. All tags were aligned with miRNA sequences present in miRBase 18.0 to identify known miRNAs in *Homosapiens* [72]. Tags not matched in miRBase were submitted to the subsequent matching steps. These unmatched tags were aligned against the sequences of non-coding RNAs (rRNA, tRNA, snRNA, snoRNA) available on Rfam [73] GenBank noncoding RNA database (http://www.ncbi.nlm.nih.gov/), repeats database [74], coding region of reference genome [75] and piRNA database (downloaded from NCBI Nucleotide database) to classify these tags into other non-coding small RNA, mRNA, genomic repeats, or unclassified tags if they were not assigned to any of the above databases.

### Prediction of Novel miRNAs

Unclassified tags consisting of at least 45 reads were retained and processed for novel miRNA prediction. The cutoff of 45 reads was chosen to avoid false positive by discarding candidate novel miRNA with low abundance. Mireap was used to predict novel miRNAs (http://sourceforge.net/projects/mireap/). The prediction following rules: 1) Minimal miRNA sequence length is 18nt; 2) Maximal miRNA sequence length is 26nt; 3) Maximal free energy allowed for a miRNA precursor is -18 kcal/mol; 4) Maximal space between miRNA and miRNA* is 35nt; 5) Maximal bulge of miRNA and miRNA* is 4. The secondary structures of the potential miRNA precursors were predicted by RNA fold (http://rna.tbi.univie.ac.at/).

### Prediction of miRNA Targets

The putative targets of selected miRNAs were predicted by miRanda [76]. The prediction followed the following rules: 1) Perfect match at the seed region (2-8nt from the 5′ end of miRNA); 2) The minimum free energy (MFE) of the miRNA/target duplex should be smaller than -20 Kcal/mol; 3) The total score for a miRNA-mRNA pairs should be greater than 140.

### GO Analysis of the Predicted miRNA Target Genes

The predicted target genes of miRNAs were subjected to the analysis of Gene Ontology terms (Ashburner, *et al*., 2000)_ENREF_8. The target genes were mapped to the GO annotation dataset, and the enriched biological processes were extracted using the Hypergeometric test.

To calculate the enrichment ratio and p-value for GO analysis, we defined N as total number of genes annotated by GO in whole genome, n as total number of genes annotated by a specific GO term in whole genome, M as total number of genes annotated by GO in predicted miRNA targets, and m as total number of genes annotated by a specific GO term in predicted miRNA targets.



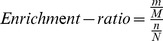
.



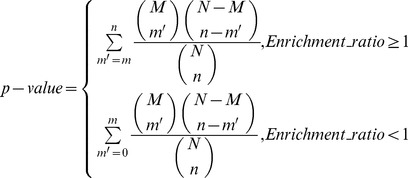
.

A GO term was identified as key term when its ratio of enrichment >2 and *p*-value <0.05.

### Pathway Analysis of the Predicted miRNA Target Genes

The target genes were mapped to the signaling pathway annotation dataset downloaded from KEGG [77]. The Fisher’s exact test on hypergeometric distribution was used to extract the enriched pathway.

To calculate the enrichment ratio and p-value for pathway analysis, we defined N as total number of genes participating in all the pathways in whole genome, n as total number of genes participating in a specific pathway in whole genome, M as total number of genes enriched in GO terms and participating in all the pathways, and m as total number of genes enriched in GO terms and participating in a specific pathway.

The formulas of enrichment calculation are the same as GO analysis of the predicted miRNA target genes.

A pathway identified when the ratio of enrichment was >1.5 and *p*-value is <0.05.

### miRNA Quantification by SYBR Green

Quantification of the mature miRNAs was performed by real-time qRT-PCR using an Applied Biosystems StepOne™ Real-Time PCR System (Applied Biosystems, Foster city, California, USA) and a SYBR premix Ex TaqTM II kit (Takara) with the primers listed in Table S13. The reactions were performed in a 48-well optical plate at 95°C for 30 s, followed by 40 cycles of 95°C for 5 s and 60°C for 31 s. All reactions were run in triplicates. After reaction, the threshold cycle (Ct) was determined by using the default threshold settings. The Ct was defined as the fractional cycle number at which the fluorescence passed a fixed threshold. The expression level of snRNA U6 was used as an internal reference.

## Supporting Information

Figure S1 Distribution of small RNAs among different categories.
(TIF)

Figure S2 Length distribution of Solexa reads. Length of unique small RNA sequencing tags. The occurrence of each unique tag was counted to reflect relative expression level and only tags in the range of 11 to 33 nt were considered.
(TIF)

Figure S3 Homologous recombination pathway was enrich based on the KEGG pathway analysis of the five novel miRNAs taget genes.
(TIF)

Figure S4 Potential targets of the most abundant piRNAs. (a) The targets of the most abundant piRNAs. (b) Classes of retrotransposons. (c) Kinds of Non-LTR retrotransposons.
(TIF)

Table S1 Statistic results of sequencing reads.
(PDF)

Table S2 The list of miRNAs and piRNAs in our data.
(XLSX)

Table S3 The expression of top five most abundant novel miRNAs.
(PDF)

Table S4 The putative target genes of top 10 abundant know miRNA.
(XLSX)

Table S5 The most enriched pathways in predicted miRNA ta rgets for top 10 abundant known miRNAs (p<0.05, E-ratio >2).
(PDF)

Table S6 The putative target genes of 5 abundant novel miRNAs.
(XLS)

Table S7 The top 10 most enriched GO terms of biological processes, molecular functions and cellular components in predicted targets of 5 abundant novel miRNAs.
(PDF)

Table S8 The most enriched pathways in predicted miRNA targets for top abundant novel miRNAs (p<0.05, E-ratio >2).
(PDF)

Table S9 The detail description of the miRNA-mRNA pairs.
(XLS)

Table S10 piRNAs with >1000 reads map uniquely using Blat within TDRG1.
(PDF)

Table S11 piRNAs with >500 reads map uniquely within CYP19A1.
(PDF)

Table S12 All miRNAs with greater than 10% of reads arising from possible editing events.
(XLSX)

Table S13 Primers used for the quantification of miRNAs.
(PDF)
