# Supplementary figures and images for: MicroRNA and piRNA Profiles in Normal Human Testis Detected by Next Generation Sequencing
Source: PLoS One. 2013 Jun 24;8(6):e66809. doi: 10.1371/journal.pone.0066809 (PMC3691314; doi:10.1371/journal.pone.0066809)

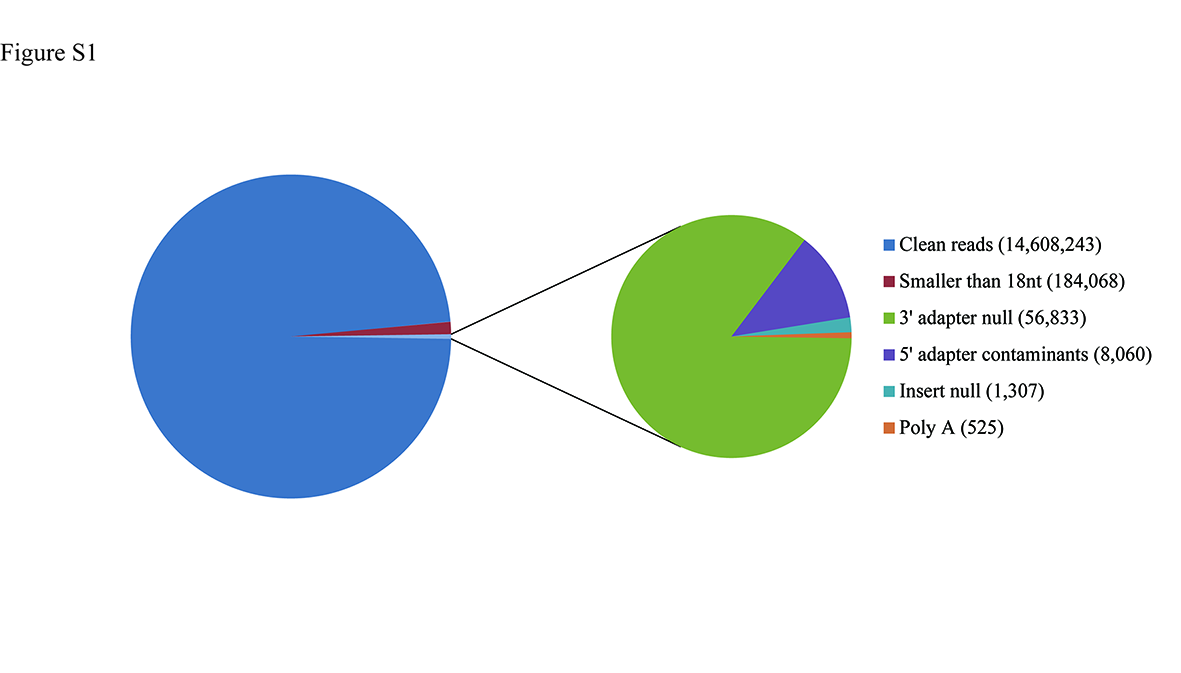

Supplement: Figure S1 — Distribution of small RNAs among different categories. (TIF) [file pone.0066809.s001.tif]

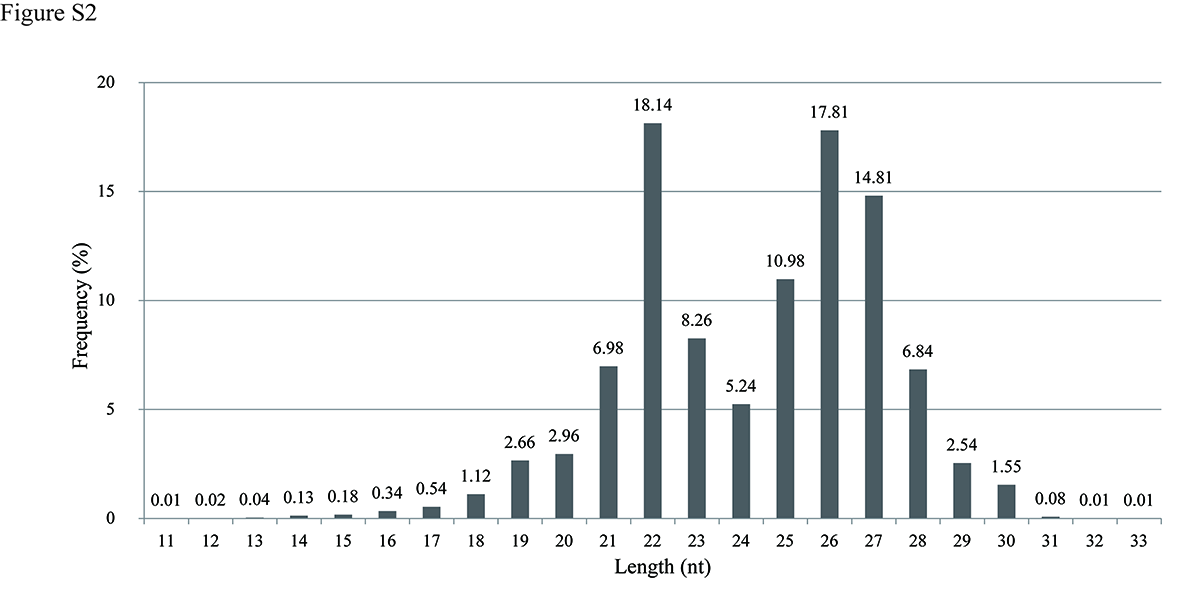

Supplement: Figure S2 — Length distribution of Solexa reads. Length of unique small RNA sequencing tags. The occurrence of each unique tag was counted to reflect relative expression level and only tags in the range of 11 to 33 nt were considered. (TIF) [file pone.0066809.s002.tif]

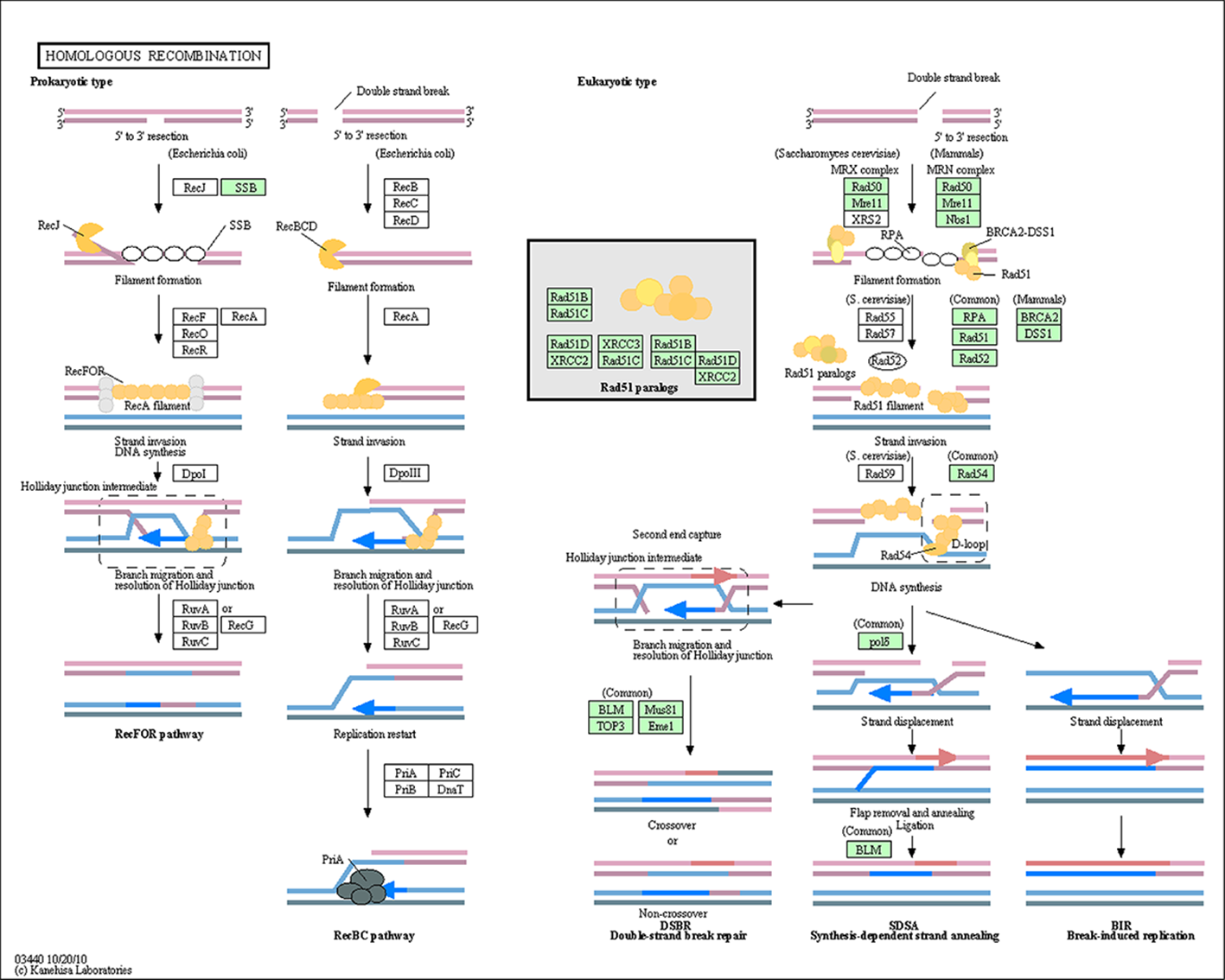

Supplement: Figure S3 — Homologous recombination pathway was enrich based on the KEGG pathway analysis of the five novel miRNAs taget genes. (TIF) [file pone.0066809.s003.tif]

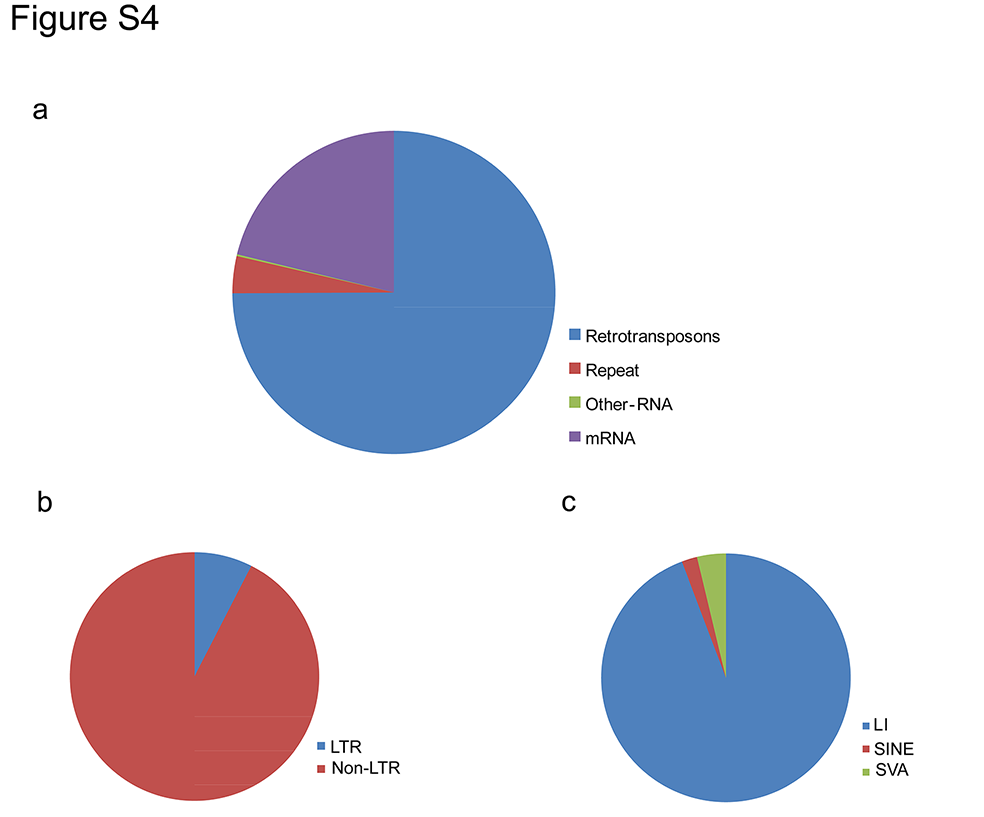

Supplement: Figure S4 — Potential targets of the most abundant piRNAs. (a) The targets of the most abundant piRNAs. (b) Classes of retrotransposons. (c) Kinds of Non-LTR retrotransposons. (TIF) [file pone.0066809.s004.tif]
