# Supplementary material for: MicroRNA and piRNA Profiles in Normal Human Testis Detected by Next Generation Sequencing
Source: PLoS One. 2013 Jun 24;8(6):e66809. doi: 10.1371/journal.pone.0066809 (PMC3691314; doi:10.1371/journal.pone.0066809)
Supplement: Table S1 — Statistic results of sequencing reads. (PDF) [file pone.0066809.s005.pdf]

Table S1. Statistic results of sequencing reads.

Type	Counts	%
Total reads	15,118,197	
Low quality reads	259,170	1.714
High quality reads	14,859,027	98.286
Clean reads	14,608,234	96.627
3' adapter null	56,833	0.376
Insert null	1,307	0.009
5' adapter contaminants	8,060	0.053
Smaller than 18 nt	184,068	1.218
Poly A	525	0.003
