# Supplementary material for: MicroRNA and piRNA Profiles in Normal Human Testis Detected by Next Generation Sequencing
Source: PLoS One. 2013 Jun 24;8(6):e66809. doi: 10.1371/journal.pone.0066809 (PMC3691314; doi:10.1371/journal.pone.0066809)
Supplement: Table S3 — The expression of top five most abundant novel miRNAs. (PDF) [file pone.0066809.s007.pdf]

Table S3. The expression of top five most abundant novel miRNAs.

Mature ID	Mature		Most abundant tag		Chromosome location	Note
	Sequence	Counts	Sequence	Counts		
HT-m0117-3p	UCGGGCGGGAGUGGUGGCUUUU	784	UCGGGCGGGAGUGGUGGCUUUU	301	chr6:28918819:28918903:+	HT-m0117-3p is sense to tRNA_Lys
HT-m0016-3p	GCAGUAGAGAAAGGAAUGAA	115	GCAGUAGAGAAAGGAAUGAA	51	chr12:100562328:100562421:+	HT-m0016-3p is antisense to GOLGA2P5
HT-m0072-3p	UGAGGAAUGUUGGAGUUAGCGG	114	UGAGGAAUGUUGGAGUUAGCGG	42	chr17:28882878:28882965:-	HT-m0072-3p is overlapping to piR-51231 (TGAGGAATGTTGGAGTTAGCGG)
HT-m0041-3p	UGAAACAAGUCUGUUAGCAAAG	47	UGAAACAAGUCUGUUAGCAAAG	25	chr15:22991026:22991118:-	HT-m0041-3p is antisense to CYFIP1
HT-m0010-5p	UAAAACUAGGACUGGUGAGUGGUU	46	UAAAACUAGGACUGGUGAGUGGUU	28	chr11:117159596:117159687:+	HT-m0010-5p antisense to BACE1 mRNA
