# Supplementary material for: MicroRNA and piRNA Profiles in Normal Human Testis Detected by Next Generation Sequencing
Source: PLoS One. 2013 Jun 24;8(6):e66809. doi: 10.1371/journal.pone.0066809 (PMC3691314; doi:10.1371/journal.pone.0066809)
Supplement: Table S5 — The most enriched pathways in predicted miRNA ta rgets for top 10 abundant known miRNAs (p<0.05, E-ratio >2). (PDF) [file pone.0066809.s009.pdf]

Table S5. The most enriched pathways in predicted miRNA targets for top 10 abundant known miRNAs ($p < 0.05$, E-ratio > 2).

| KEGG pathway ID | Human testis | | Genome | | Gene Symbol | E-ratio | p-value |
|---|---|---|---|---|---|---|---|
| | Number | % | Number | % | | | |
| hsa00532 | 5 | 1.08 | 22 | 0.39 | *chst12/chst13/chst3/ust/b3gat2/* | 2.78 | 0.03 |
| hsa04930 | 9 | 1.94 | 47 | 0.83 | *socs4/irs2/socs1/adipoq/pik3ca/abcc8/cacnaie/socs3/inf/* | 2.34 | 0.01 |
| hsa04340 | 10 | 2.15 | 56 | 0.98 | *csnk1d/wnt7a/wnt9a/gas1/ptch2/csnk1g2/fbxw11/wnt2/btrc/sufu/* | 2.18 | 0.02 |
| hsa04115 | 12 | 2.58 | 68 | 1.20 | *igf1/casp3/sesn3/steap3/tsc2/bai1/ccne2/cdk6/tp73/pten/sesn2/ccne1/* | 2.16 | 0.01 |
| hsa05144 | 9 | 1.94 | 51 | 0.90 | *comp/cr1/darc/klrk1/met/tgfb2/cd81/il6/tnf/* | 2.16 | 0.03 |
| hsa05222 | 14 | 3.01 | 83 | 1.46 | *col4a6/lama5/plas4/itga2b/pik3ca/ccne2/cdk6/col4a4/ikbkg/lamb2/pten/ptgs2/ccne1/cdkn2b/* | 2.06 | 0.01 |
| hsa05410 | 14 | 3.01 | 83 | 1.46 | *cacnb4/igf1/itga7/dmd/iyga2b/cacna2d2/itgb4/myl3/ppkag1/tgfb2/il6/itga10/tnf/* | 2.06 | 0.01 |
| hsa04114 | 18 | 3.87 | 112 | 1.97 | *cpeb/igf1/anapc13/espl1/igf1r/ppp2r1b/slk/ccne2/ppp2r5d/camk2b/fbxw11/plk1/ppp2ca/rps6ka2/ ywhag/btrc/ccne1/rps6ka3* | 1.97 | 0.01 |
| hsa05414 | 14 | 3.01 | 90 | 1.58 | *cacnb4/igf1/itga7/dmd/adrb1/itga2b/cacna2d2/itgb4/myl3/pln/tgfb2/itga10/tnf/* | 1.90 | 0.02 |
| hsa05146 | 16 | 3.44 | 105 | 1.85 | *col4a6/arg2/casp3/col1a2/gnal/lama5/plcb2/serpinb9/pik3ca/col4a4/lamb2/muc2/tgfb2/actn3/il6/tnf/* | 1.86 | 0.02 |
| hsa05142 | 15 | | 102 | 1.79 | *ccl3/ccl3l1/ccl3l3/faslg/gnal/plcb2/ppp2r1b/pik3ca/ikbkg/irak4/tgfb2/ppp2ca/c1qb/il6/tnf* | 1.80 | 0.03 |
