# Supplementary material for: MicroRNA and piRNA Profiles in Normal Human Testis Detected by Next Generation Sequencing
Source: PLoS One. 2013 Jun 24;8(6):e66809. doi: 10.1371/journal.pone.0066809 (PMC3691314; doi:10.1371/journal.pone.0066809)
Supplement: Table S7 — The top 10 most enriched GO terms of biological processes, molecular functions and cellular components in predicted targets of 5 abundant novel miRNAs. (PDF) [file pone.0066809.s011.pdf]

Table S7.
The top 10 most enriched GO terms of biological processes, molecular functions and cellular components in predicted targets of 5 abundant novel miRNAs.

Description of GO term	Human testis		Genome		E-ratio	p-value
	Number	%	Number	%		
The top 10 most enriched biological processes						
Proximal/distal pattern formation (GO:0009954)	6	0.16	23	0.91	5.82	4.32E-04
B cell activation (GO:0042113)	6	0.20	29	0.91	4.62	1.61E-03
Vasculature development (GO:0001944)	6	0.20	30	0.91	4.46	1.94E-03
Activation of pro-apoptotic gene products (GO:0008633)	6	0.21	31	0.91	4.32	2.31E-03
Induction of apoptosis by intracellular signals (GO:0008629)	7	0.30	44	1.06	3.55	3.31E-03
Neuromuscular process controlling balance (GO:0050885)	6	0.27	40	0.91	3.35	8.53E-03
Odontogenesis of dentine-containing tooth (GO:0042475)	8	0.37	55	1.22	3.25	3.10E-03
T cell activation (GO:0042110)	6	0.30	44	0.91	3.04	1.35E-02
Embryonic digit morphogenesis (GO:0042733)	6	0.30	44	0.91	3.04	1.35E-02
JNK cascade (GO:0007254)	7	0.38	56	1.06	2.79	1.25E-02
The top 10 most enriched cellular components						
Synaptobrevin 2-SNAP-25-syntaxin-1a-complexin I complex (GO:0070032)	2	0.02	3	0.28	14.98	5.79E-03
Mre11 complex (GO:0030870)	2	0.03	5	0.28	8.99	1.82E-02
Pre-autophagosomal structure (GO:0000407)	2	0.04	6	0.28	7.49	2.65E-02
U7 snRNP (GO:0005683)	2	0.04	7	0.28	6.42	3.60E-02
Platelet dense tubular network membrane (GO:0031095)	2	0.04	7	0.28	6.42	3.60E-02
PTW/PP1 phosphatase complex (GO:0072357)	2	0.04	7	0.28	6.42	3.60E-02
Melanosome membrane (GO:0033162)	2	0.05	8	0.28	5.62	4.65E-02
Nuclear chromatin (GO:0000790)	11	0.39	64	1.52	3.86	1.20E-04
Chromatin (GO:0000785)	12	0.48	78	1.65	3.46	1.80E-04
Nuclear body (GO:0016604)	4	0.17	28	0.55	3.21	3.45E-02
The top 10 most enriched molecular functions						
Nucleoside kinase activity (GO:0019206)	3	0.04	6	0.42	11.18	1.63E-03
3'-phosphoadenosine 5'-phosphosulfate binding (GO:0050656)	3	0.04	6	0.42	11.18	1.63E-03
SNARE binding (GO:0000149)	4	0.11	18	0.56	4.97	7.48E-03
Galactosyltransferase activity (GO:0008378)	5	0.16	25	0.70	4.47	4.56E-03
Peptide antigen binding (GO:0042605)	3	0.09	15	0.42	4.47	2.74E-02
Syntaxin binding (GO:0019905)	4	0.13	21	0.56	4.26	1.31E-02
Oxygen binding (GO:0019825)	6	0.21	33	0.83	4.06	3.19E-03
Epidermal growth factor receptor binding (GO:0005154)	3	0.11	18	0.42	3.73	4.44E-02
Extracellular matrix binding (GO:0050840)	3	0.11	18	0.42	3.73	4.44E-02
Beta-amyloid binding (GO:0001540)	4	0.16	26	0.56	3.44	2.75E-02
