# Supplementary material for: MicroRNA and piRNA Profiles in Normal Human Testis Detected by Next Generation Sequencing
Source: PLoS One. 2013 Jun 24;8(6):e66809. doi: 10.1371/journal.pone.0066809 (PMC3691314; doi:10.1371/journal.pone.0066809)
Supplement: Table S8 — The most enriched pathways in predicted miRNA targets for top abundant novel miRNAs (p<0.05, E-ratio >2). (PDF) [file pone.0066809.s012.pdf]

Table S8. The most enriched pathways in predicted miRNA targets for top abundant novel miRNAs ($p < 0.05$, E-ratio > 2).

KEGG pathway ID	Human testis		Genome		Gene Symbol	E-ratio	p-value
	Number	%	Number	%			
hsa04320	4	0.42	24	1.74	*ets1/notch3/fmn2/kras/*	4.12	1.53E-02
hsa03440	4	0.49	28	1.74	*top3b/mus81/Amre11a/nbn/*	3.53	2.59E-02
hsa00512	4	0.53	30	1.74	*galnt9/st3gal2/b4galt5/c1gal1/*	3.30	3.25E-02
hsa04720	8	1.23	70	3.48	*adcy1/c alml5/crebbp/grin1/grm1/rps6ka2/kras/ppp3ca/*	2.83	7.81E-03
hsa04070	8	1.37	78	3.48	*calml5/cdipt/impa2/pik3r2/itpk1/inpp4a/pikfyve/dgkq/*	2.54	1.45E-02
hsa04146	8	1.39	79	3.48	*crat/pxmp/acsl3/nudt12/pecr/pex11a/pex1/pex3/*	2.50	1.56E-02
hsa04520	7	1.28	73	3.04	*actn1/crebbp/igf1r/fer/fgfr1/samd3/yes1/*	2.37	2.96E-02
hsa04662	7	1.32	75	3.04	*cd81/ikbkg/pik3r2/fcgr2b/kras/nfat5/ppp3ca/*	2.31	3.37E-02
hsa04914	8	1.51	86	3.48	*adcy1/anacp2/igf1r/pik3r2/rps6ka2/mapk14/kras/mapk8/*	2.30	2.47E-02
hsa04020	15	3.11	177	6.52	*adcy1/atp2a3/atp2b2/cacan1a/cacan1i/calml5/chrna7/grin1/grm/p2rx3 /sla8a2/chrm3/erbb3/phkg2/ppp3ca/*	2.10	1.10E-02
hsa04660	9	1.90	108	3.91	*cd3g/cd8a/ikbkg/pdcd1/pik3r2/mapk14/kras/nfat5/ppp3ca/*	2.06	4.36E-02
hsa05200	21	5.70	324	9.13	*crebbp/dapk1/ets1/fzd2/gli2/igf1r/ikbkg/ntrk1/plas4/pik3r2/stat5b/ wnt6/itga2b/kars/mapk8/runx1t1/casp3/fgfr1/ralb/samd3/xiap/*	1.60	4.39E-02
