# Supplementary material for: MicroRNA and piRNA Profiles in Normal Human Testis Detected by Next Generation Sequencing
Source: PLoS One. 2013 Jun 24;8(6):e66809. doi: 10.1371/journal.pone.0066809 (PMC3691314; doi:10.1371/journal.pone.0066809)
Supplement: Table S10 — piRNAs with >1000 reads map uniquely using Blat within TDRG1. (PDF) [file pone.0066809.s014.pdf]

Table S10. piRNAs with >1000 reads map uniquely using Blat within TDRG1

Reads	Length	Counts	Sequence	Chromosome location
t0000248	27	2610	TCAGGACTGCTGAAAGAATGAAGAAGA	chr6:40346167-40346193 +
t0000099	27	6717	TGAAAGAATGAAGAAGAACTTACTTGG	chr6:40346177-40346203 +
t0000268	27	2424	AAAGAATGAAGAAGAACTTACTTGGCC	chr6:40346179-40346205 +
t0000093	27	6886	AAGAATGAAGAAGAACTTACTTGGCCT	chr6:40346180-40346206 +
t0000109	26	6111	AAGAATGAAGAAGAACTTACTTGGCC	chr6:40346180-40346205 +
t0000407	26	1600	TGAAGAAGAACTTACTTGGCCTAGGA	chr6:40346185-40346210 +
t0000527	25	1178	TAGAGAACGGAGCGCACTTTCACTT	chr6:40346217-40346241 +
t0000196	24	3519	TAGGTGTGGAGCTTCCCGACCGGC	chr6:40346309-40346332 +
t0000013	25	68013	TAGGTGTGGAGCTTCCCGACCGGCT	chr6:40346309-40346333 +
t0000046	26	15766	TAGGTGTGGAGCTTCCCGACCGGCTG	chr6:40346309-40346334 +
t0000364	27	1783	TCTCTGAAACCTCCAGCATTTGTGCCC	chr6:40346576-40346602 +
t0000395	26	1649	TAGAATCAGTCAAGAGGAATTGGGCC	chr6:40347350-40347375 +
