# Supplementary material for: MicroRNA and piRNA Profiles in Normal Human Testis Detected by Next Generation Sequencing
Source: PLoS One. 2013 Jun 24;8(6):e66809. doi: 10.1371/journal.pone.0066809 (PMC3691314; doi:10.1371/journal.pone.0066809)
Supplement: Table S11 — piRNAs with >500 reads map uniquely within CYP19A1. (PDF) [file pone.0066809.s015.pdf]

Table S11. piRNAs with >500 reads map uniquely within CYP19A1

Reads	Length	Counts	Sequence	Chromosome location
t0000263	26	2453	TTTGAGCTGGACCTCGAAAGATGGCA	chr15:51594905-51594930+
t0000347	28	1862	TGGGAATGTGAAGCCTGGAGCAGGAGTC	chr15:51581611-51581638+
t0000351	25	1845	TTTGAGCTGGACCTCGAAAGATGGC	chr15:51594906-51594930+
t0000352	28	1844	TAGCTTCTCTGCCTTGCATCTGACTGAC	chr15:51584226-51584253+
t0000603	27	988	TAGCTTCTCTGCCTTGCATCTGACTGA	chr15:51584227-51584253+
t0000826	26	696	GTTTGAGCTGGACCTCGAAAGATGGC	chr15:51594906-51594931+
