# Supplementary material for: MicroRNA and piRNA Profiles in Normal Human Testis Detected by Next Generation Sequencing
Source: PLoS One. 2013 Jun 24;8(6):e66809. doi: 10.1371/journal.pone.0066809 (PMC3691314; doi:10.1371/journal.pone.0066809)
Supplement: Table S13 — Primers used for the quantification of miRNAs. (PDF) [file pone.0066809.s017.pdf]

Table S13. Primers used for the quantification of miRNAs.

Name	Reverse transcript stem-loop primer	Gene specific Primer for Realtime-PCR	Universal Primer
hsa-let-7f-5p	GTCGTATCCAGTGCGTGTCGTGGAGTCGGCAATTGCACTGGATACGACAACTATA	GCGTGAGGTAGTAGATTGT	CAGTGCGTGTCGTGGAGT
hsa-let-7a-5p	GTCGTATCCAGTGCGTGTCGTGGAGTCGGCAATTGCACTGGATACGACAACTATA	ACGTGAGGTAGTAGGTTGT	CAGTGCGTGTCGTGGAGT
hsa-miR-34c-5p	GTCGTATCCAGTGCGTGTCGTGGAGTCGGCAATTGCACTGGATACGACGCAATCA	GCAGGCAGTGTAGTTAGC	CAGTGCGTGTCGTGGAGT
hsa-miR-202-5p	GTCGTATCCAGTGCGTGTCGTGGAGTCGGCAATTGCACTGGATACGACCAAAGAA	GCGTTCCTATGCATATACTT	CAGTGCGTGTCGTGGAGT
hsa-let-7c	GTCGTATCCAGTGCGTGTCGTGGAGTCGGCAATTGCACTGGATACGACAACCATA	GCGTGAGGTAGTAGGTTG	CAGTGCGTGTCGTGGAGT
hsa-miR-10b-5p	GTCGTATCCAGTGCGTGTCGTGGAGTCGGCAATTGCACTGGATACGACCACAAAT	GGTACCCTGTAGAACCGA	CAGTGCGTGTCGTGGAGT
hsa-miR-514b-5p	GTCGTATCCAGTGCGTGTCGTGGAGTCGGCAATTGCACTGGATACGACATGATTG	GCTTCTCAAGAGGGAGGC	CAGTGCGTGTCGTGGAGT
hsa-miR-34b-5p	GTCGTATCCAGTGCGTGTCGTGGAGTCGGCAATTGCACTGGATACGACCAATCAG	GCGTAGGCAGTGTCATTAG	CAGTGCGTGTCGTGGAGT
hsa-miR-15b-5p	GTCGTATCCAGTGCGTGTCGTGGAGTCGGCAATTGCACTGGATACGACTGTAAAC	GCGTAGCAGCACATCATGG	CAGTGCGTGTCGTGGAGT
hsa-miR-27b-3p	GTCGTATCCAGTGCGTGTCGTGGAGTCGGCAATTGCACTGGATACGACGCAGAAC	GCGTTCACAGTGGCTAAG	CAGTGCGTGTCGTGGAGT
hsa-miR-134	GTCGTATCCAGTGCGTGTCGTGGAGTCGGCAATTGCACTGGATACGACCCCCTCT	GCGTGTGACTGGTTGACC	CAGTGCGTGTCGTGGAGT
hsa-miR-15a-5p	GTCGTATCCAGTGCGTGTCGTGGAGTCGGCAATTGCACTGGATACGACCACAAAC	GCGTAGCAGCACATAATGG	CAGTGCGTGTCGTGGAGT
hsa-miR-889	GTCGTATCCAGTGCGTGTCGTGGAGTCGGCAATTGCACTGGATACGACACAATGG	GCCTTAATATCGGACAACC	CAGTGCGTGTCGTGGAGT
hsa-miR-506-3p	GTCGTATCCAGTGCGTGTCGTGGAGTCGGCAATTGCACTGGATACGACTCTACTC	CGTAAGGCACCCTTCTG	CAGTGCGTGTCGTGGAGT
hsa-miR-17-5p	GTCGTATCCAGTGCGTGTCGTGGAGTCGGCAATTGCACTGGATACGACCTACCTG	ACGCAAAGTGCTTACAGTG	CAGTGCGTGTCGTGGAGT
HT-m0117_3p	GTCGTATCCAGTGCGTGTCGTGGAGTCGGCAATTGCACTGGATACGACAAAAGCC	TCGGGCGGGAGTGGT	CAGTGCGTGTCGTGGAGT
HT-m0016_3p	GTCGTATCCAGTGCGTGTCGTGGAGTCGGCAATTGCACTGGATACGACTTCATTC	GCGGCAGTAGAGAAAGG	CAGTGCGTGTCGTGGAGT
HT-m0072_3p	GTCGTATCCAGTGCGTGTCGTGGAGTCGGCAATTGCACTGGATACGACCCGCTAA	GCGTGAGGAATGTTGGAGT	CAGTGCGTGTCGTGGAGT
HT-m0041_3p	GTCGTATCCAGTGCGTGTCGTGGAGTCGGCAATTGCACTGGATACGACCTTTGCT	GGCTGAAACAAGTCTGTTAG	CAGTGCGTGTCGTGGAGT
HT-m0010_5p	GTCGTATCCAGTGCGTGTCGTGGAGTCGGCAATTGCACTGGATACGACAACCACT	AGGTAAAACTAGGACTGGTG	CAGTGCGTGTCGTGGAGT
U6	CGCTTCACGAATTTGCGTGTCAT	fw GCTTCGGCAGCACATATACTAAAAT	rw CGCTTCACGAATTTGCGTGTCAT
